# Grating-lobe-free optical phased array with 2-D circular sparse array aperture and high-efficiency phase calibration

**DOI:** 10.1515/nanoph-2023-0519

**Published:** 2024-01-02

**Authors:** Daixin Lian, Shi Zhao, Wenlei Li, Jingye Chen, Daoxin Dai, Yaocheng Shi

**Affiliations:** State Key Laboratory for Modern Optical Instrumentation, Center for Optical & Electromagnetic Research, College of Optical Science and Engineering, International Research Center for Advanced Photonics, Zhejiang University, Zijingang Campus, Hangzhou 310058, China; Ningbo Innovation Center, College of Optical Science and Engineering, Zhejiang University, Ningbo Campus, Ningbo 315100, China; Jiaxing Key Laboratory of Photonic Sensing & Intelligent Imaging, Jiaxing 314000, China; Intelligent Optics & Photonics Research Center, Jiaxing Research Institute Zhejiang University, Jiaxing 314000, China; Ningbo Research Institute, Zhejiang University, Ningbo 315100, China.

**Keywords:** silicon, optical phased array, sparse array, phase calibration

## Abstract

An optical phased array (OPA) with 2-D circular sparse array aperture has been proposed and demonstrated in the silicon integrated photonic platform. The sparse distribution of the antenna array can realize no grating lobes in 2-D full field of view (FOV). To achieve fast and accurate phase calibration for OPA, an improved rotating element electric field vector algorithm based on golden section search method (GSS-REV) has also been proposed and verified. The 32-element antenna sparse distribution of the proposed OPA is designed and fabricated. A far-field beam steering measurement across 20° × 20° range features the side lobe suppression ratio (SLSR) of larger than 4.81 dB and a full width at half-maximum (FWHM) of approximately 0.63° × 0.59°. The resolvable points are derived to be ∼1076. The OPA chip has also been demonstrated on range measurement with frequency-modulated continuous-wave (FMCW) system.

## 1 Introduction

Light detection and ranging (LIDAR) technology is one of the most crucial sensor technologies which plays an important role in the field of autonomous driving, artificially intelligent robots, and free-space communications [1], [2], [3], [4]. Recently, the silicon integrated photonic platform has achieved compact and powerful on-chip optical devices, which promotes the development of solid-state LIDAR with high integration and miniaturization [5], [6], [7], [8].

In the past decade, the optical phased array (OPA) based on silicon integrated photonic platform has become a hot topic [6], [7], [8], [9], [10], [11], [12]. At present, two categories of architectures of 2-D OPA have been proposed: 1-D antenna arrays that utilize phase and wavelength tuning, and 2-D antenna array with pure phase tuning. The main problem of the former architecture is the relatively small steering range which is limited by the bandwidth of the tunable laser, resulting in a large steering range inevitably increases system cost [4], [6], [13]. While the steering range for the 2-D antenna array with pure phase tuning is limited by the severe grating lobes. Recently, 2-D sparse antenna arrangement methods have also been reported [14], [15]. Due to the minimum spacing limited by the antenna size, grating lobes still cannot be eliminated with rectangular sparse apertures, which leads to a limited field of view (FOV). Another method is to use uniform circular aperture architecture [16], [17], [18]. However, OPA with uniform circular aperture still has relatively uniform and large grating lobes. For example, by neglecting the influence of the antenna components, the highest grating lobes level of −1.24 dB can be achieved in 16-element OPA based on uniformly single circular aperture, which is presented in Figure S1(b) of the Supplementary Material. Reasonable sparsity under the original layout will break the circular symmetry and achieve lower background noise.

In addition, phase calibration is usually necessary due to the optical waveguide length difference and the unavoidable wave-guide width deviation during fabrication, leading to the random phase difference among array elements [8], [19]. Many algorithms such as genetic algorithm (GA) [20], simulated annealing (SA) algorithm [21] and stochastic parallel gradient descent (SPGD) algorithm [22] have been proposed to calibrate the phase error. However, these algorithms have the disadvantage of local convergence or time-consuming.

In this work, we propose and experimentally demonstrate a 2-D circular sparse OPA on silicon-on-insulator (SOI) platform. The sparse array aperture has been optimized by the genetic algorithm to increase the suppression ratio of side lobe (SLSR) and reduce the system complexity. Moreover, we propose an improved rotating element electric field vector algorithm based on golden section search method (GSS-REV) for phase calibration, which dramatically improves the efficiency by 36 times of modified rotating element electric field vector (mREV) and 39 times of genetic algorithm (GA). The range measurement with frequency-modulated continuous-wave (FMCW) system for the fabricated OPA chip has also been demonstrated.

## 2 Optimization of the 2-D circular sparse element distribution

**Figure 1:**
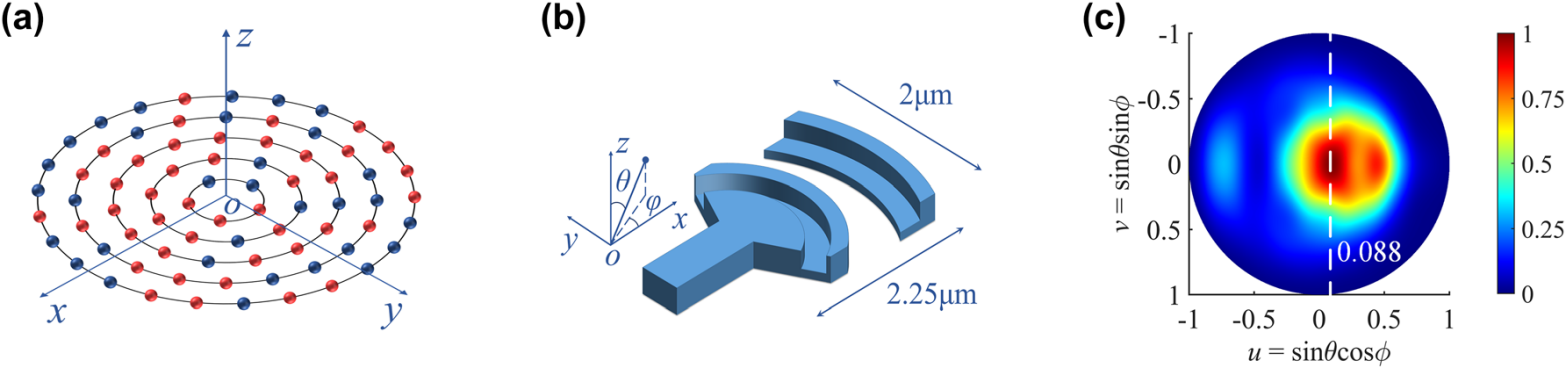
Illustration of the 2-D circular sparse element distribution. (a) Schematic of a multi-circular sparse aperture OPA with retained (blue) and removed (red) radiating elements; (b) 3-D schematic of the silicon-based surface-emitting antenna with L-shaped radiating segments; (c) far-field radiation pattern at 1550 nm wavelength for the single antenna element.

A schematic diagram of the proposed circular sparse aperture OPA is presented in Figure 1(a). The circular array consists of *M* concentric rings of linear increasing radius (*r*_*m*_ = *m*·*r*_1_) and spheres with linearly increasing numbers (*N*_*m*_ = *m*·*N*_1_) on the innermost to outermost ring, where the blue spheres represent the retained radiating antenna elements and red are the removed elements. As shown in Figure 1(b), the size of the single radiating antenna element with L-shaped radiating segments is 2.25 μm × 2 μm. Figure 1(c) is the simulation of the far-field pattern for the antenna element working at 1550 nm wavelength.

In the initial layout, we assume that the radiating antenna element can be treated as an isotropic point radiator, the array factor of the multi-circular OPA with (*u*_0_, *v*_0_) as the main lobe can be expressed as [18]:

(1) $$\begin{array}{ll} F_{\left(u,v,u_{0},v_{0}\right)} & =\overset{M}{\underset{m=1}{\sum }}\overset{N_{m}}{\underset{n=1}{\sum }}E_{m,n}e^{\left[jkr_{m}\left(\cos\varphi_{m,n}\left(u-u_{0}\right)+\sin\varphi_{m,n}\left(v-v_{0}\right)\right)\right]} \end{array}$$

where *E*_*m*,*n*_ is the E-field intensity for the particular element, *k* is the wave vector, *φ*_*m*,*n*_ = 2π(*n* − 1/2)/*N*_*m*_ is the angular position of the *n*th element on the *m*th ring, *u* and *v* are calculated by *u* = sin*θ*cos*φ* and *v* = sin*θ*sin*φ*, *θ* is the elevation angle and *φ* is the azimuthal angle.

In order to effectively eliminate the grating lobes and improve SLSR, we propose to apply genetic algorithm [23], [24] to search for an optimal element distribution under the initial circular distribution. Here, we define the binary initial population *f* to represent the state matrix of elements, of which each individual contains 32 “ones” and 43 “zeros” randomly distributed determining the position coordinates of the 32 retained elements. After multiple generations of selection, crossover, and mutation operations, the element distribution corresponding to the optimal figure of merit (FOM) is output when the termination condition is satisfied. The FOM is calculated by 10log10(*P*_main_/*P*_side_), here, *P*_main_ is the power of the main lobe in the direction of (0.088, 0) and *P*_side_ is the maximum side-lobe power within a 60° FOV centered on the main lobe. The details are shown in Equation (S1) of the Supplementary Material.

**Figure 2:**
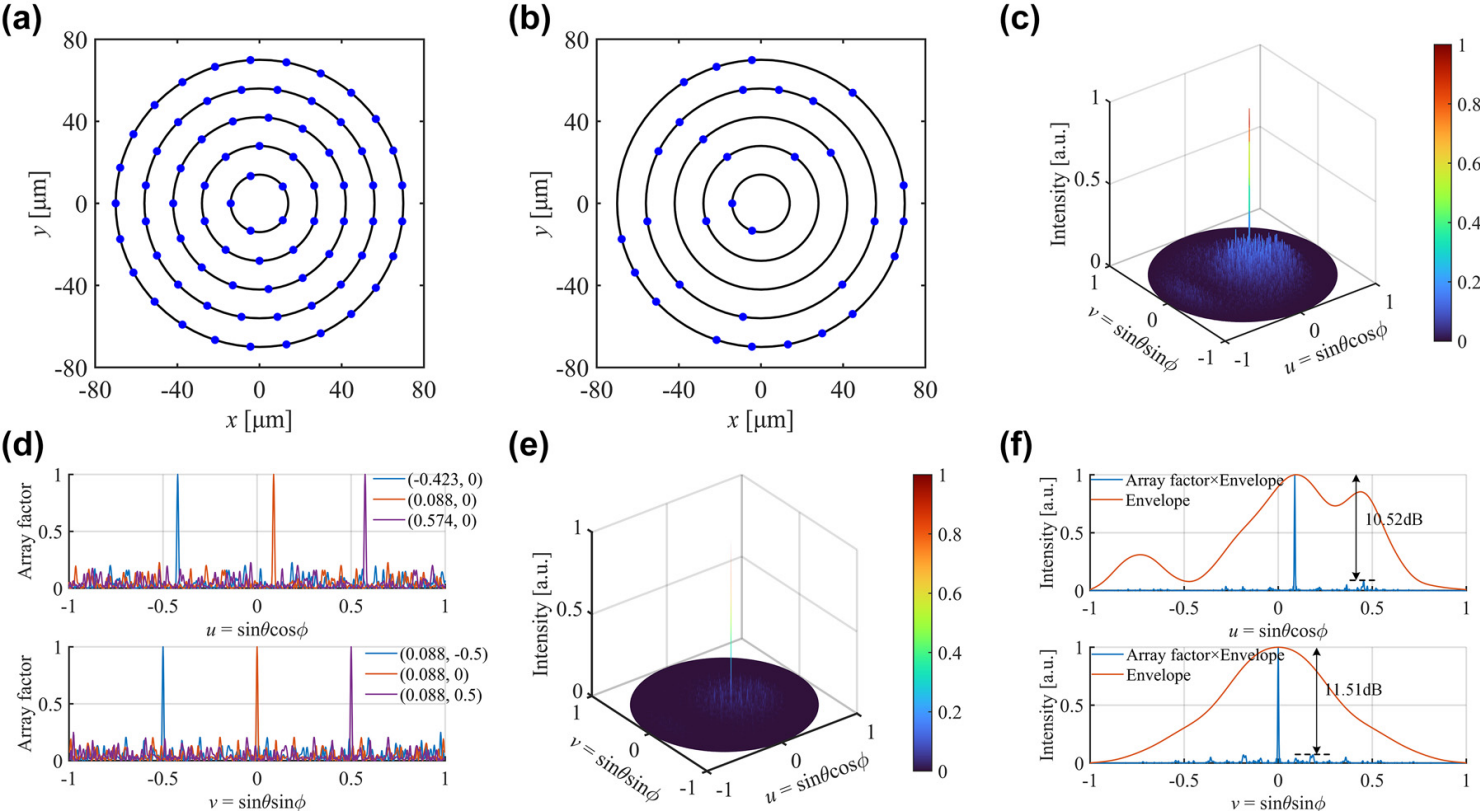
Optimization results for 32-element and 128-element circular sparse array. (a) Initial antenna element distribution; (b) sparse antenna distribution with 32-element; (c) 2-D far-field intensity pattern on the *u*–*v* plane with the distribution of (b); (d) 1-D cross-sections of the far-field array factor patterns in both *u* (*v* = 0 plane) and *v* (*u* = 0.088 plane) directions with the distribution of (b); (e) 2-D far-field intensity pattern on the *u*–*v* plane of 128-element sparse distribution based on circular aperture; (f) 1-D cross-sections of the intensity pattern along two orthogonal directions of the main lobe for (e).

The element distribution before and after optimization are shown in Figure 2(a) and (b), respectively. The normalized 2-D far-field intensity on the *u*–*v* plane under the optimal distribution shown in Figure 2(c) demonstrates a 0.58° × 0.52° FWHM of the main lobe and a SLSR of about 7.68 dB. Neglecting the influence of the diffraction envelope, the maximum side lobe has little change and no grating lobes present in the whole FOV during the process of the beam deflecting towards both axes, which is shown in Figure 2(d). Besides, the ratio of power in the main lobe and the total power of the optimized array is approximately double of the initial array. The results are presented in the Supplementary Material .

The 128-element circular sparse OPA is also designed to achieve a higher SLSR through the same optimization steps, which has 10 rings with a total of 275 initial elements (the elements on each ring increase linearly from 5 to 50). The radius of rings increases linearly from 14 μm to 140 μm. Figure 2(e) and (f) show a far-field beam with a small FWHM of 0.33° × 0.31° and a SLSR up to 10.52 dB.

## 3 GSS-REV algorithm for phase calibration

Due to the unequal optical waveguide length and the unavoidable random variation of the waveguide width introduced by the fabrication deviations, the light emitted from the antenna array will have a random phase distribution, generating a random far-field beam pattern [8]. Adaptive algorithms such as GA or SPGD for phase calibration are easily trapped at local optima when the scale of OPA increases because of the non-convexity of the optimization [6], [25].

**Figure 3:**
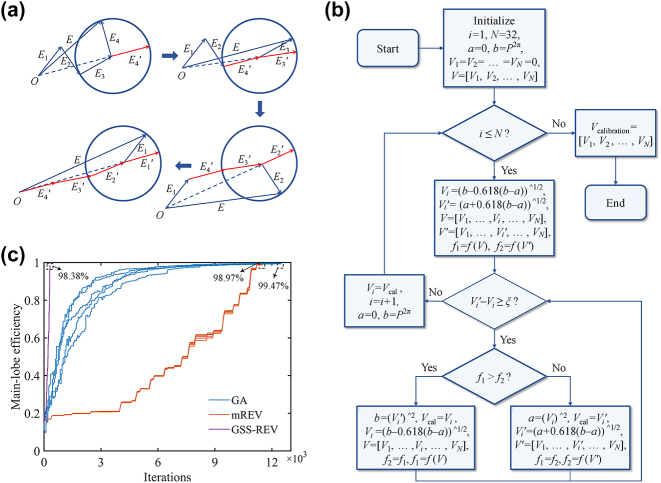
Illustration of GSS-REV algorithm. (a) Schematic diagram of GSS-REV algorithm principle; (b) flow chart of the proposed GSS-REV algorithm; (c) comparison of the performance among GA, mREV and GSS-REV algorithm.

We proposed an improved REV algorithm based on the golden section search method (GSS-REV). The algorithm inherits the advantage of nearly global optimization of mREV algorithm and greatly improves the efficiency. The calibration principle of GSS-REV and mREV algorithm are the same, which is shown in Figure 3(a). All vectors need to be redirected to maximize the strength of superimposed vector [26]. Figure 3(b) shows the flow chart of GSS-REV algorithm. We set the starting point of the optimization interval to 0, and the ending point to *P* ^2π^, corresponding to the modulation power required for 2π phase change. Then the interval length is continuously reduced by 0.618 times until the algorithm reaches convergence accuracy to search for the calibration voltage of each channel in sequence.

Three algorithms are used for performance comparison in the same initial phase distribution, which are separately run 5 times. The main-lobe efficiency (ME) is defined as the ratio of the main lobe power after a certain round of iteration and that without theoretical phase error. We assume that the phase resolution is 1° during the phase calibration. And a Gaussian distribution error within the range of 0.1 % peak main lobe intensity is introduced in far-field optical power measurement data to simulate the effect of power measurement inaccuracy, stray ambient light, et al. The population size and evolutionary generations are set to 50 and 250 in GA. For GSS-REV algorithm, the convergence accuracy *ξ* is set to 0.074, representing a distinguishable accuracy of 1° for two trial points and 10 iterations for each channel calibration. Figure 3(c) shows that among the three algorithms, GA achieves a highest ME of averagely 99.47 % with large iterations of 12,500. The algorithm of mREV requires 360 iterations for each channel; the average ME is 98.97 %. GSS-REV algorithm has the best performance since it takes only 10 iterations to find the optimal solution for single channel, and ultimately achieves a ME of 98.38 % averagely by totally 320 iterations, which significantly improves the efficiency by 36 times and 39 times at the cost of little calibration accuracy loss compared with mREV and GA, respectively.

## 4 Fabrication and characterization

**Figure 4:**
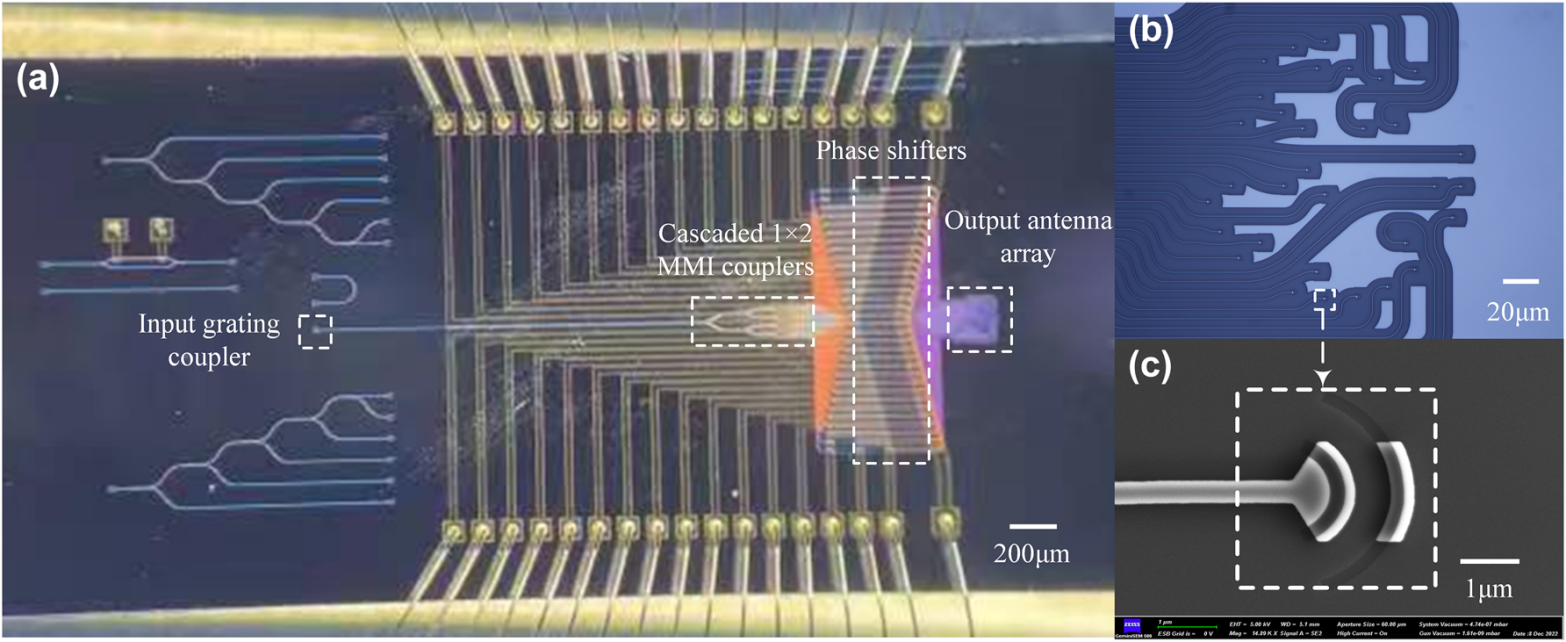
Physical photos of the proposed OPA chip. (a) Microscope image of the fabricated device; (b) enlarged image of the output antenna array; (c) SEM image of single radiating antenna.

The 32-element OPA with circular sparse antenna is then fabricated on the SOI wafer with a 220 nm thick silicon layer and a 2 μm thick buried silicon oxide layer. It consists of input grating coupler, cascaded multimode interference (MMI) coupler, 32 independent thermo-optical phase shifters, and output antenna array with a whole circular aperture of 140 μm in diameter. The microscope image of the fabricated OPA chip is shown in Figure 4(a) and the enlarged image of the output antenna array is shown in Figure 4(b), which is the same as Figure 2(b). Figure 4(c) shows the scanning electron microscope (SEM) image of the single antenna element with a 220 nm deep etching step and a 150 nm shallow etching step.

The Mach–Zehnder interferometer (MZI) structure is utilized to characterize the performance of the phase shifter and determine the parameters required for phase calibration of OPA. The measurement results are shown in Figure S8 of the Supplementary Material.

**Figure 5:**
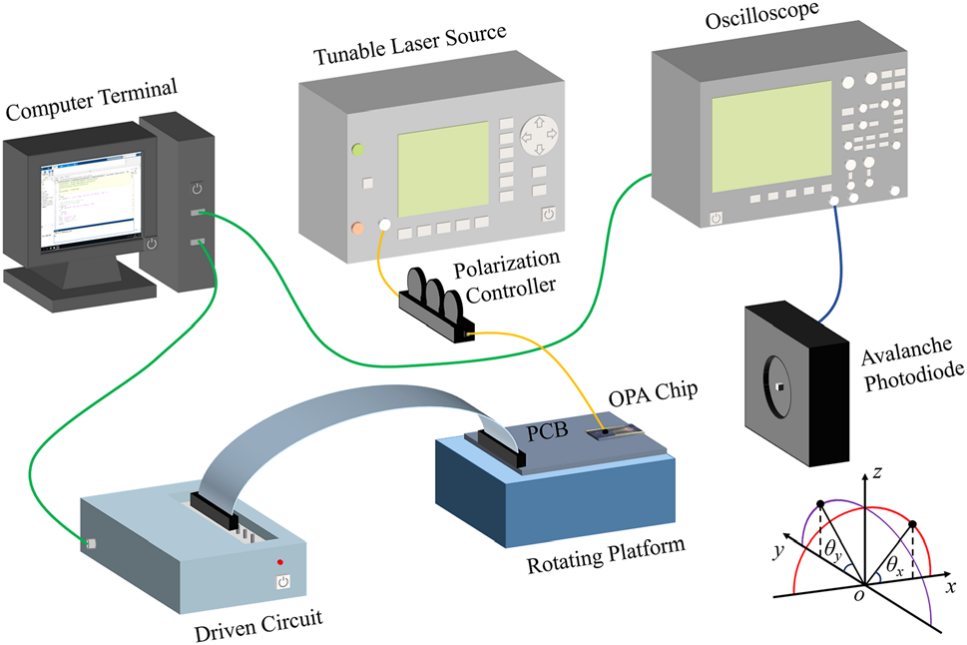
Schematic diagram of OPA phase calibration system.

To decrease the influence of obvious noise and thermal crosstalk during the phase calibration process, two rounds of calibration are performed, which takes around 40 s to calibrate single angle for our hardware implementation due to the relatively large time delay of communication and feedback between instruments. The power optimization interval [*a*, *b*] for each channel is set to [*P*_rand_, *P*_rand_ + *P* ^2π^]. Here, *P*_rand_ is a randomly generated small power value to allow a random distribution of voltages for all channels as much as possible during the calibration process. The phase calibration setup is shown in Figure 5. A tunable laser source (Keysight, 81,940A) is used as the input source at 1550 nm wavelength with 13 dBm output power. The OPA chip is placed on the rotating platform and the far-field main lobe power of the OPA is monitored by an avalanche photodiode (Thorlabs, APD430C/M) and fed back to the computer terminal through oscilloscope (Keysight, DSO7032B) to form a closed-loop system.

**Figure 6:**
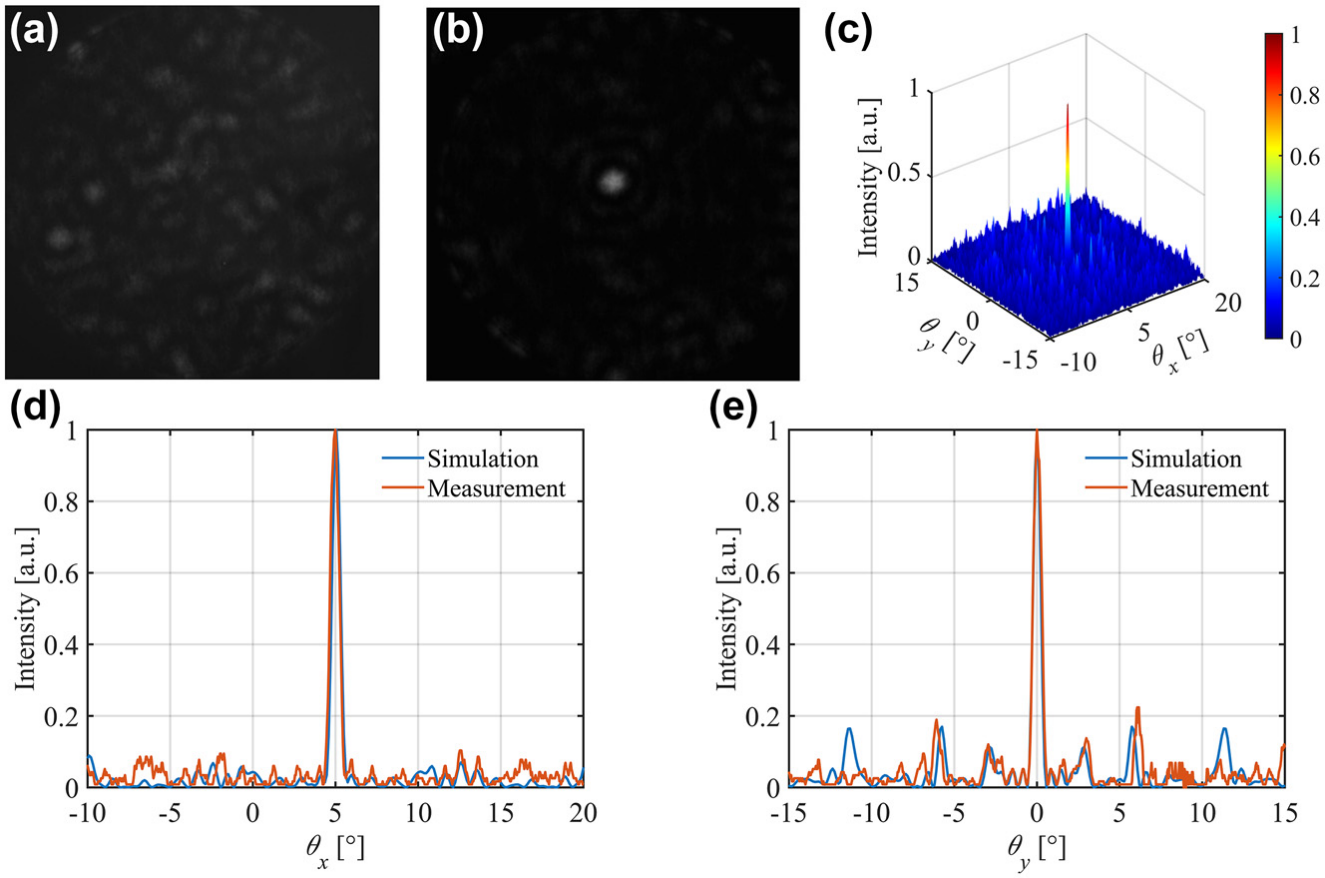
Measurement results for single steering angle of (5°, 0°). Uncalibrated (a) and calibrated (b) far-field observed image. (c) Measured 2-D far-field intensity pattern in a 30° × 30° FOV. 1-D cross-sections of the far-field intensity pattern in (d) along *θ*_*x*_ (*θ*_*y*_ = 0° plane) and (e) along *θ*_*y*_ (*θ*_*x*_ = 5° plane) directions.

**Figure 7:**
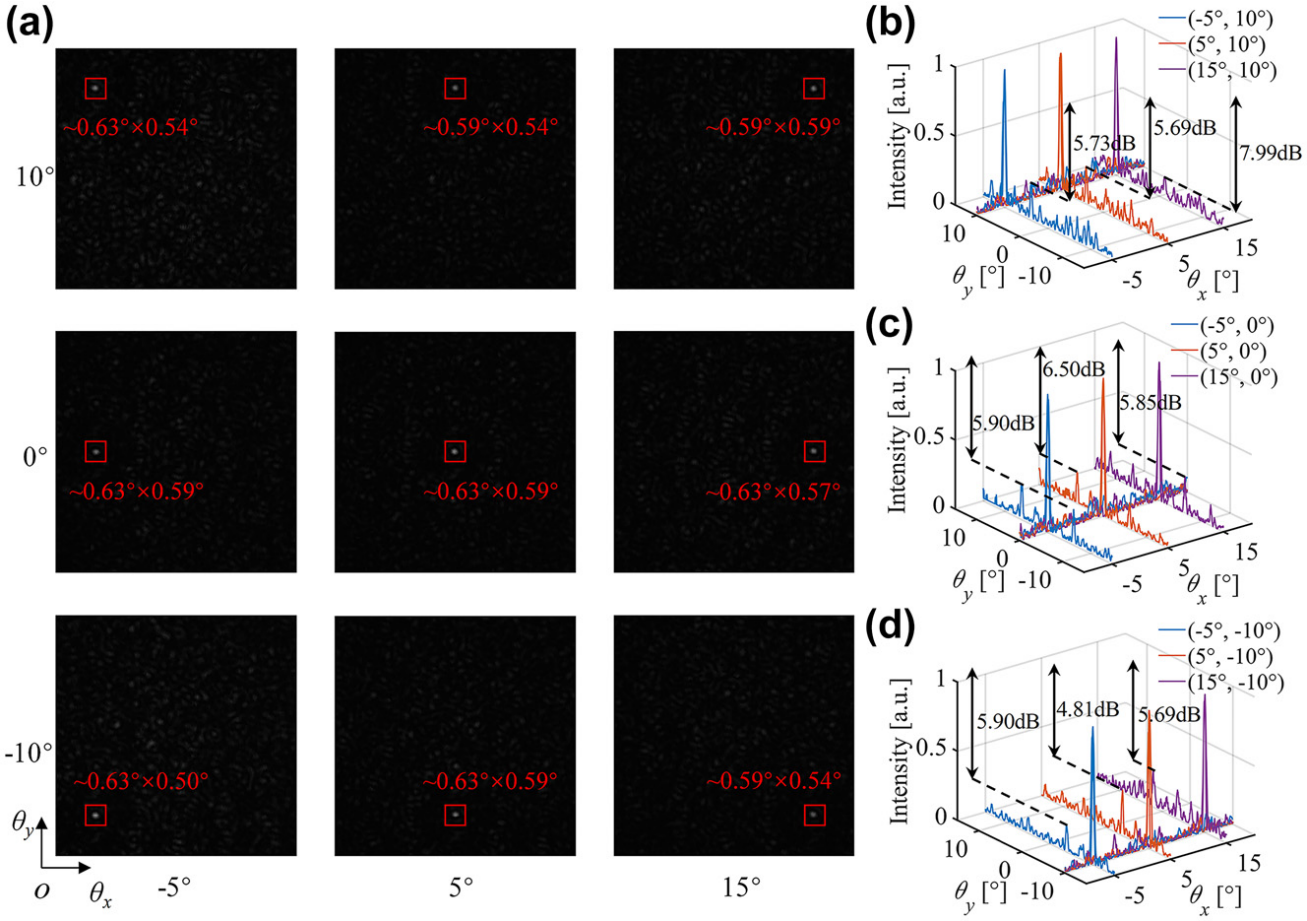
Measurement results for 9 steering angles in a 20° × 20° range. (a) Camera image of far-field beam steering in different directions. 1-D cross-sections of the intensity pattern along two orthogonal directions of the main lobe in (b) *θ*_*y*_ = 10° plane, (c) *θ*_*y*_ = 0° plane and (d) *θ*_*y*_ = −10° plane.

The infrared charge coupled device (CCD) camera (C12741-03, HAMAMATSU) is used to capture the far-field images of OPA. We define the coordinate axes as shown in Figure 5. Figure 6(a) and (b) show the far-field images with the main lobe of (5°, 0°) in the FOV of the infrared CCD before and after calibration, respectively. The measured normalized 2-D beam pattern with a SLSR of 6.50 dB for the optimized direction is shown in Figure 6(c). The comparison between the simulation and measurement of 1-D cross-sections of the beam intensity pattern in *θ*_*x*_ and *θ*_*y*_ are shown in Figure 6(d) and (e), respectively. The closeness between the simulation curve and the measurement curve verifies the feasibility of the OPA chip and accuracy of GSS-REV algorithm for phase calibration and the slight difference could be attributed to the fabrication error and the limited applied voltage accuracy of the control circuit. The far-field patterns of different steering angles are also obtained and analysed. Figure 7(a) shows the far-field beam images for different steering angles, which demonstrates a maximum FWHM of approximately 0.63° × 0.59° and totally approximately 1076 resolvable points in a 20° × 20° steering range. Due to the decrease of the antenna envelope strength, the side-lobe level rises when the beam deflects to a large angle and the minimum SLSR is demonstrated to be 4.81 dB according to Figure 7(b)–(d).

**Figure 8:**
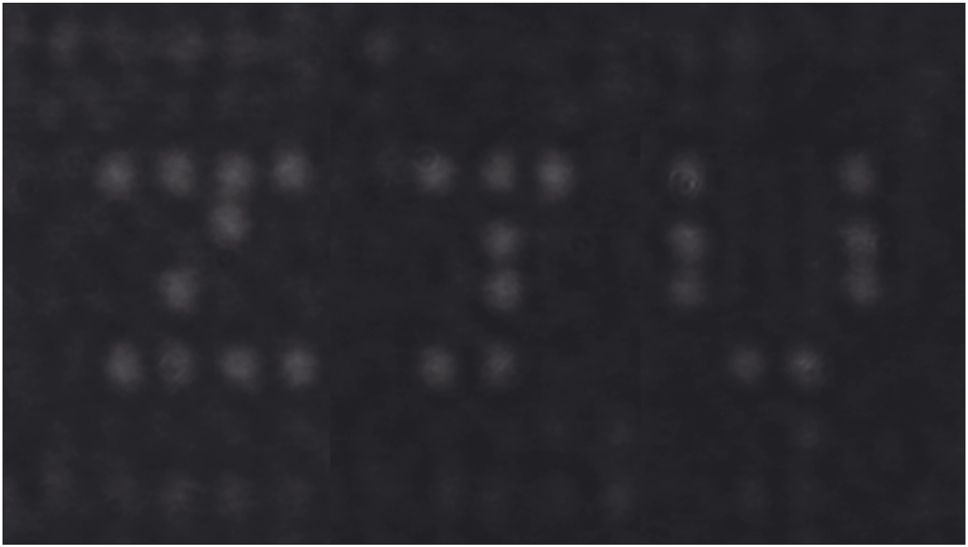
Patterns of “*Z*”, “*J*”, “*U*” formed by different steering points.

To further demonstrate the 2-D beam steering ability of our proposed OPA, as shown in Figure 8, we control the steering angle of the far-field beam so that the superposition of these steering points presents the character of “*Z*”, “*J*”, and “*U*”.

**Figure 9:**
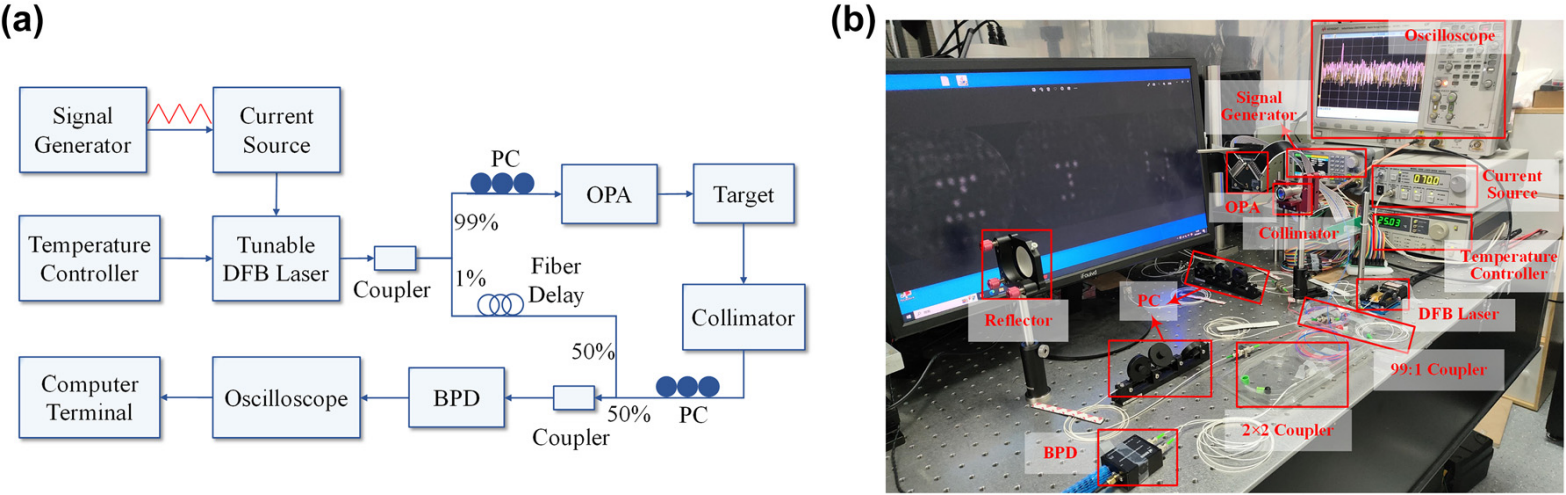
Configuration of the FMCW system based on the proposed OPA. (a) Schematic diagram and (b) setup of FMCW system for free-space LIDAR range measurement.

In addition, the free-space LIDAR range measurement has been demonstrated with the fabricated OPA and the FMCW system. The setup of FMCW system is shown in Figure 9. The OPA chip is used in conjunction with two free-space lenses, one for reflecting the transmit beam emitted from the chip and the other for collimating the receive beam and focusing the beam onto the fibre. The local oscillator and received optical signal are mixed and subsequently enter the balanced photodetector to generate the beat frequency signal which is displayed on the oscilloscope screen through the fast Fourier transform (FFT).

Generally, there are two main methods to generate a frequency-modulated signal. They are external modulation and internal modulation, respectively. We use the internal modulation method to generate a linear frequency modulation signal for the distributed feedback (DFB) laser since it is the simplest way to achieve frequency modulation and avoid expensive devices like microwave source. However, extra calibration is needed as the dependence between the drive current and laser output frequency is not linear. The gradient descent algorithm is employed to calibrate the drive signal [27]. The increment of the drive voltage in each segment generated by the iteration (*n*) can be expressed as follows:

(2) $$\begin{array}{ll} {\left[\Delta u\left(t_{i}\right)\right]}_{n+1} & ={\left[\Delta u\left(t_{i}\right)\right]}_{n}\cdot \gamma_{\text{ideal}}/\gamma_{\text{actual}}\\ & ={\left[\Delta u\left(t_{i}\right)\right]}_{n}\cdot \gamma_{\text{ideal}}/\left[f\left(t_{i+1}\right)-f\left(t_{i}\right)/\Delta t\right] \end{array}$$

where *f*(*t*_*i*_) is the sweep frequency which is calculated by time domain waveform of beat frequency through Hilbert transform, the ideal sweep slope *γ*_ideal_ is calculated as 2 *B*/*T*, *B* is the bandwidth of the sweep frequency and *T* is the period of the triangle waveform.

**Figure 10:**
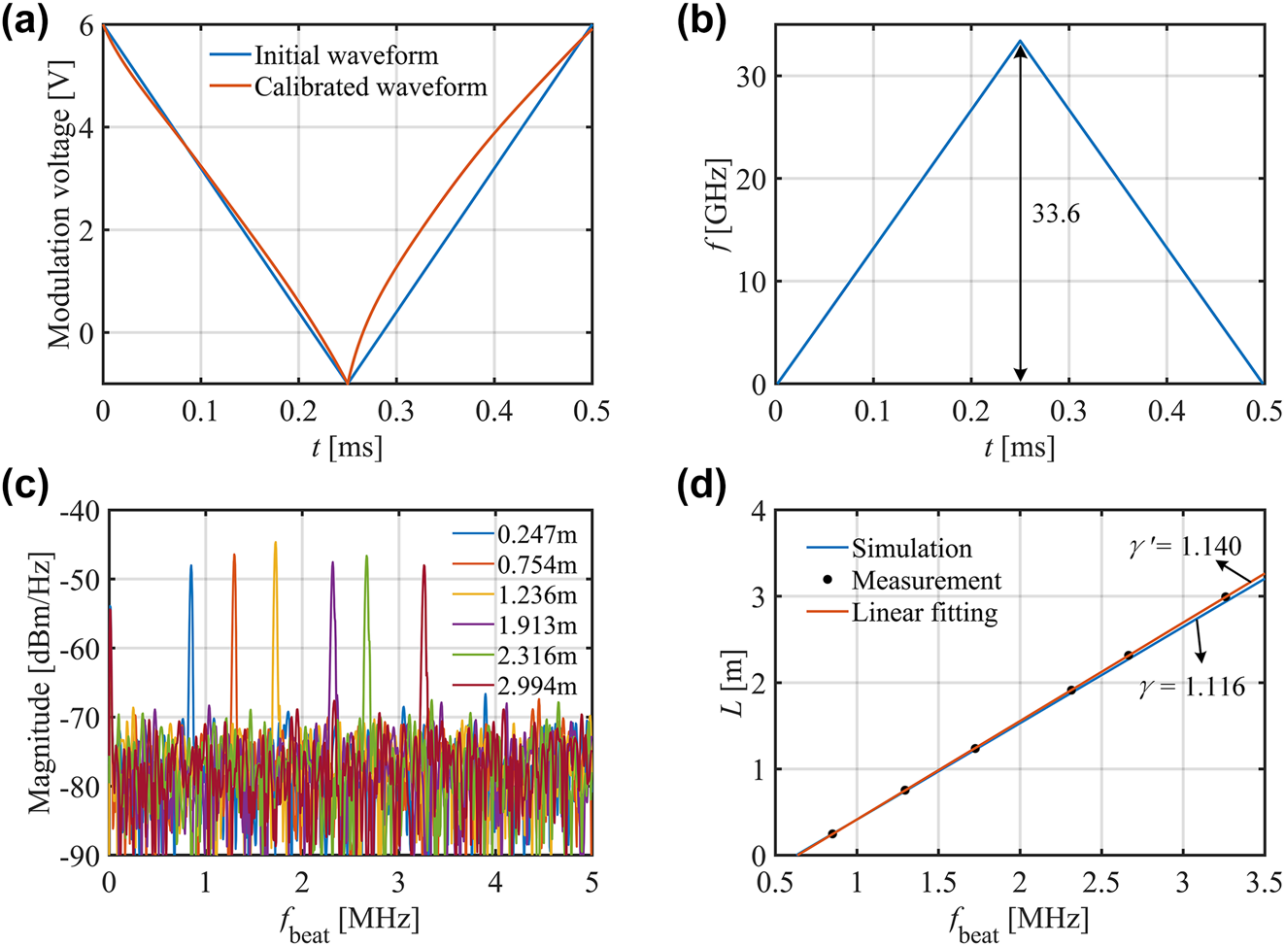
Free-space LIDAR range measurement. (a) Modulation voltage waveform before and after calibration; (b) sweep frequency under the calibrated voltage of signal generator; (c) measured beat frequency spectra for different distance; (d) comparison of simulation and measurement of the curve of distance *L* and beat frequency *f*_beat_.

The voltage waveform is obtained through 10 iterations, as shown in Figure 10(a). And Figure 10(b) shows the corresponding sweep frequency bandwidth *B* of 33.6 GHz. As shown in Figure 10(c), the measured beat frequency spectra for different distances show the beat frequencies at different distances and present a good signal-to-noise ratio of larger than 20 dB. As shown in Figure 10(d), we linearly fit the measured distance and beat frequency. The coefficient of determination of 0.9999 indicates perfect linearity. Compared with the theoretical slope *γ* of 1.116 m/MHz, the fitting slope *γ*′ is calculated to be 1.140 m/MHz. The difference of the slope could be attributed to the slight nonlinearity of the sweep frequency and the impact of background noise.

## 5 Conclusions

In summary, we have proposed and experimentally demonstrated a silicon photonic OPA with circular sparse array aperture, which efficiently overcomes the grating lobes issue caused by the large element spacing. The novel sparse distribution of the antenna array can realize no grating lobes in 2-D full FOV. The GSS-REV algorithm is also proposed to overcome the defect of local convergence and improve the efficiency of the OPA phase calibration. During measurement, 640 iterations are performed for single steering angle and the far-field intensity value of the target angle can be completely convergent. Furthermore, we combine the fabricated OPA chip with FMCW system to demonstrate Free-space LIDAR range measurement. Theoretical analysis also shows that small FWHM and large SLSR can be achieved by further increasing the number of the antenna.

## Supplementary Material

Supplementary Material Details
