# Supplementary material for: Grating-lobe-free optical phased array with 2-D circular sparse array aperture and high-efficiency phase calibration
Source: Nanophotonics. 2024 Jan 2;13(1):29–37. doi: 10.1515/nanoph-2023-0519 (PMC11501881; doi:10.1515/nanoph-2023-0519)
Supplement: Supplementary file 1 — Supplementary Material Details [file j_nanoph-2023-0519_suppl_001.docx]

Supplementary Materials for: Grating-lobe-free optical phased array with 2-D circular sparse array aperture and high-efficiency phase calibration

Daixin Lian, Shi Zhao, Wenlei Li, Jingye Chen, Daoxin Dai and Yaocheng Shi*

Centre for Optical and Electromagnetic Research, State Key Laboratory for Modern Optical Instrumentation, Zhejiang University, Zijingang Campus, Hangzhou 310058, China

*Address correspondence to: yaocheng@zju.edu.cn.

**Contents**

1. Analysis of the OPA with 2-D uniform circular aperture

2. Method of the sparse element distribution based on genetic algorithm

3. Principle of golden section search method

4. Characterization of the phase modulator

5. Calculation of the calibrated voltage for the particular steering angle

6. Range measurement based on the FMCW system

**1. Analysis of the OPA with 2-D uniform circular aperture**

There is a significant difference between the far-field of the OPA based on 2-D uniform rectangular aperture and that based on 2-D uniform circular aperture. Figure S1(a) and S1(b) respectively show the far-field intensity of these two distribution. The rectangular aperture has strong grating lobes because of the large element spacing, which severely limit the field of view (FOV) of OPA. Due to the sinusoidal variation of element spacing in the *x* and *y* directions of uniform circular aperture, no periodic grating lobes exist in these two orthogonal directions of the far field of this type of OPA. However, the grating lobes are distributed on the concentric circles centered on the main lobe. In this example, the highest grating lobe level can reach approximately -1.24 dB indicating a high level of background noise in the far-field.


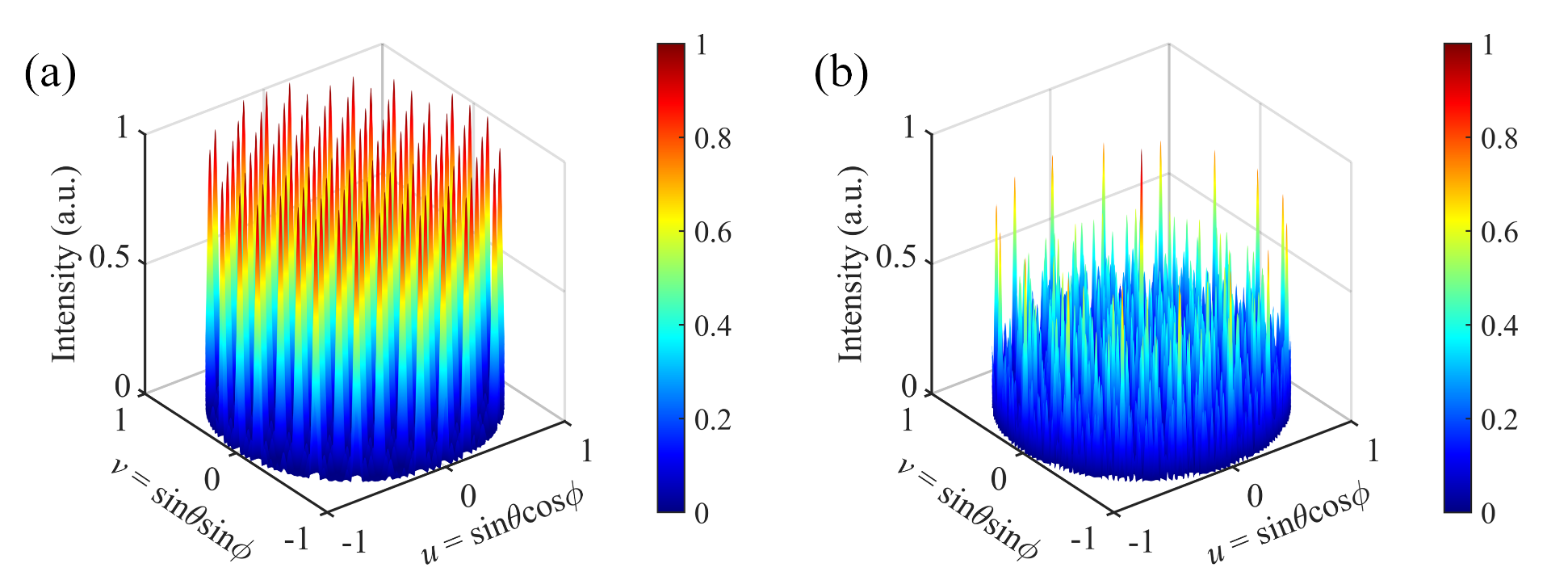


**Figure S1:** Far-field of 16-element OPA based on (a) 4×4 uniform rectangular aperture with element spacing of 8 μm and (b) uniform single circular aperture with the radius of 20.4 μm.

To achieve a better performance of suppressing the side-lobe level of the OPA. The initial distribution of elements is important. Here, the circular array with single and multiple rings are simulated, respectively. As shown in Figure S2(a) and S2(b), multiple rings may achieve a destructive interference for grating lobes generated by singe ring when the spacing of adjacent elements larger than 1/2 wavelength at the cost of an increase in overall side-lobe level.


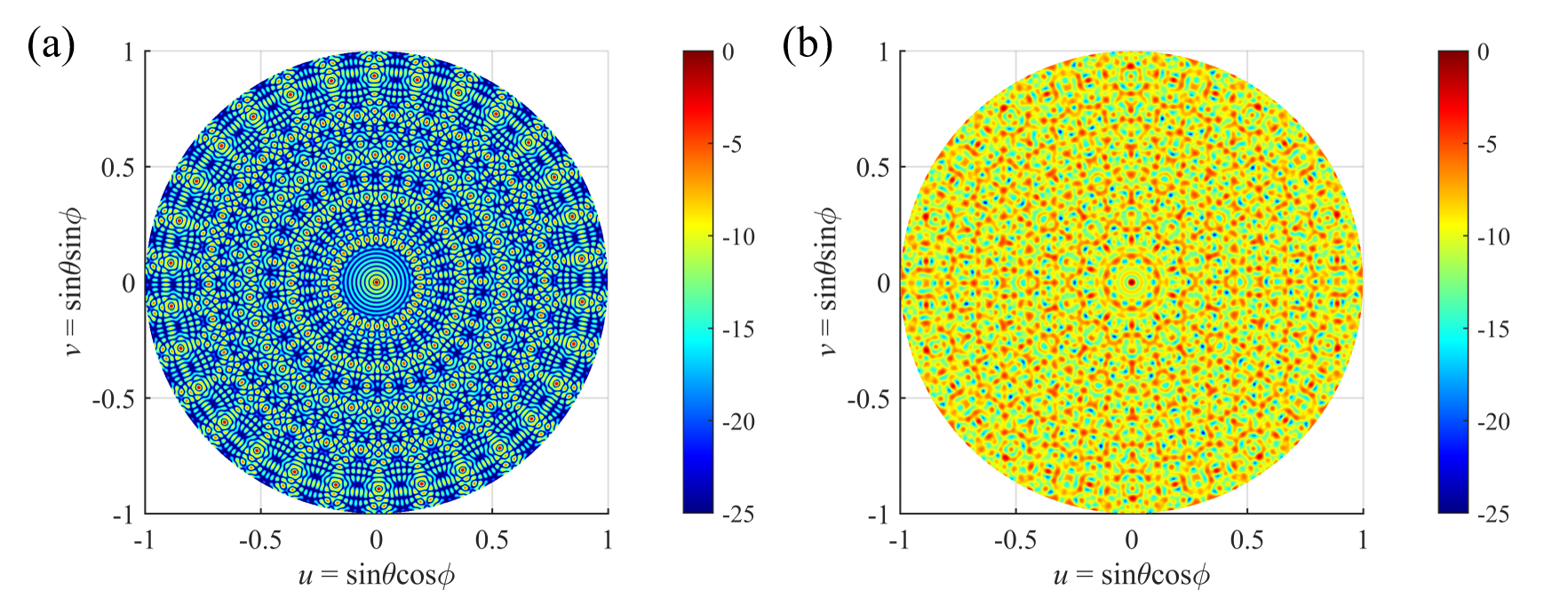


**Figure S2:** Far-field of 30-element OPA based on (a) uniform single circular aperture with the radius of 42 μm and (b) uniform multiple circular aperture with 3 rings, the radii are 14, 28, 42 μm, respectively.

For the initial OPA layout, we choose a scheme which is to apply 5,10,15,20,25 elements respectively for the 5 rings, it takes into account the changes between odd and even numbers of all rings. To reduce the complexity of optimization, the radius of the first ring and the radial increments of ring diameters are set as a constant (14 μm) since it is a suitable distance for arranging antenna elements. Besides, we have simulated different distributions with different initial azimuthal angle (*θ_i_*, *i*=1~5) of the first element on each ring. The results in Figure S3 indicate the similar side-lobe level. Therefore, we set $\theta_{i}=\pi/(10\cdot i),\text{ }i\text{=1\textasciitilde5}$ in the following optimization for better routing.


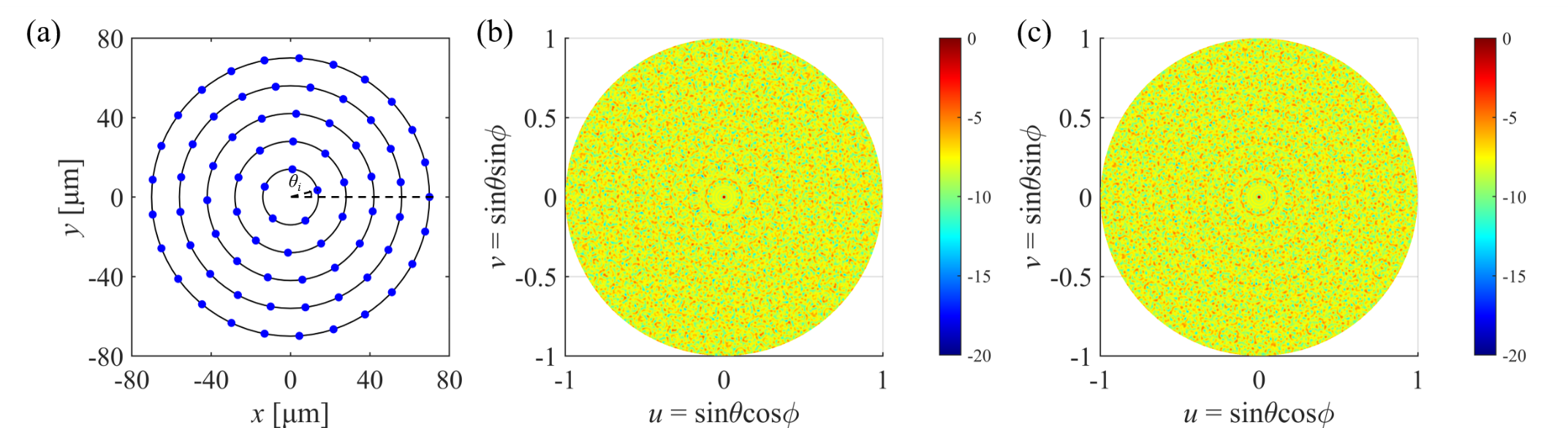


**Figure S3:** (a) Distribution of elements on 5 rings, (b) and (c) are far-field pattern with random *θ_i_*, *i*=1~5.

**2. Method of the sparse element distribution based on genetic algorithm**

To increase the side-lobe suppression ratio (SLSR) of the optical phased array (OPA) base on circular aperture and reduce the complexity of OPA system, genetic algorithm (GA) is applied to remove certain antenna elements from the original circular layout. The figure of merit (FOM) of algorithm is defined by:

$$\begin{matrix} \begin{matrix} \text{FOM}=10\log10\left(\frac{P_{\text{main}}}{P_{\text{side}}}\right)=20\log10\left(\frac{F_{\left(u_{0},v_{0},u_{0},v_{0}\right)}}{\max\left(F_{\left(u,v,u_{0},v_{0}\right)}\right)}\right), \\ u_{0}=0.088,\text{ }v_{0}=0,\text{ }u\in\left\{u_{0}\pm\sin(30^{\circ})\right\},\text{ }v\in\left\{v_{0}\pm\sin(30^{\circ})\right\} \end{matrix} & (\text{S}1) \end{matrix}$$

where, the *F* function is the array factor defined by Equation (1). (*u*, *v*) is the projection in the *x* and *y* directions of an angular vector located in the unit spherical coordinate system within a 60° FOV centered on the main lobe of (0.088, 0). The basic steps of GA include encoding, decoding, selection, crossover, mutation, the above steps are repeated to gradually approximate to the optimal figure of merit (FOM) value. The algorithm flow chart is shown in Figure S4(a). Here, the population size *NP* is set to be 50, the evolutionary generation *G* to be 100, the number of rings *M* to be 5, the number of elements on the first ring *N*_1_ to be 5, the radius of the first ring *r*_1_ to be 14 μm, and operating wavelength *λ* to be 1550 nm. As shown in Figure S4(b), The FOM is increased from the initial 3.43 dB to 6.01 dB after optimization. And the corresponding optimal distribution of 32 elements is shown in Figure S4(c). And the far-field intensity patterns of the initial and optimized distribution are presented in Figure S5(a) and S5(b), and the 1-D cross sections of intensity along two directions are shown in Figure S5(c) and S5(d), which verifies the much lower intensity of the side lobes in the optimized array and no grating lobes in the full FOV.


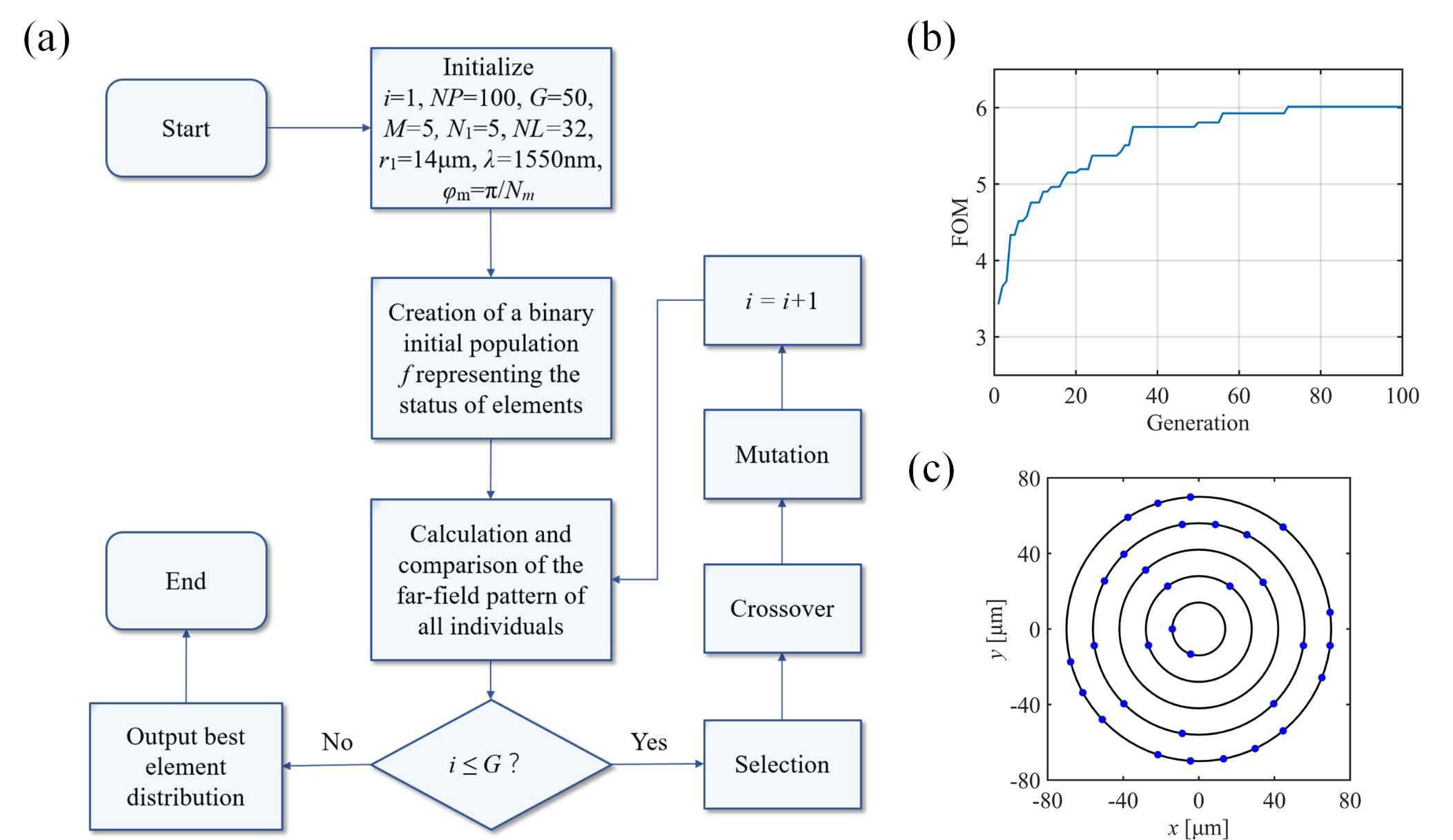


**Figure S4:** Optimization for the 32-element sparse circular array. (a) Flow chart of genetic algorithm for the optimization of the element distribution. (b) Convergence curve for the FOM in each generation during the optimization. (c) Distribution of the optimized 32 antennas.


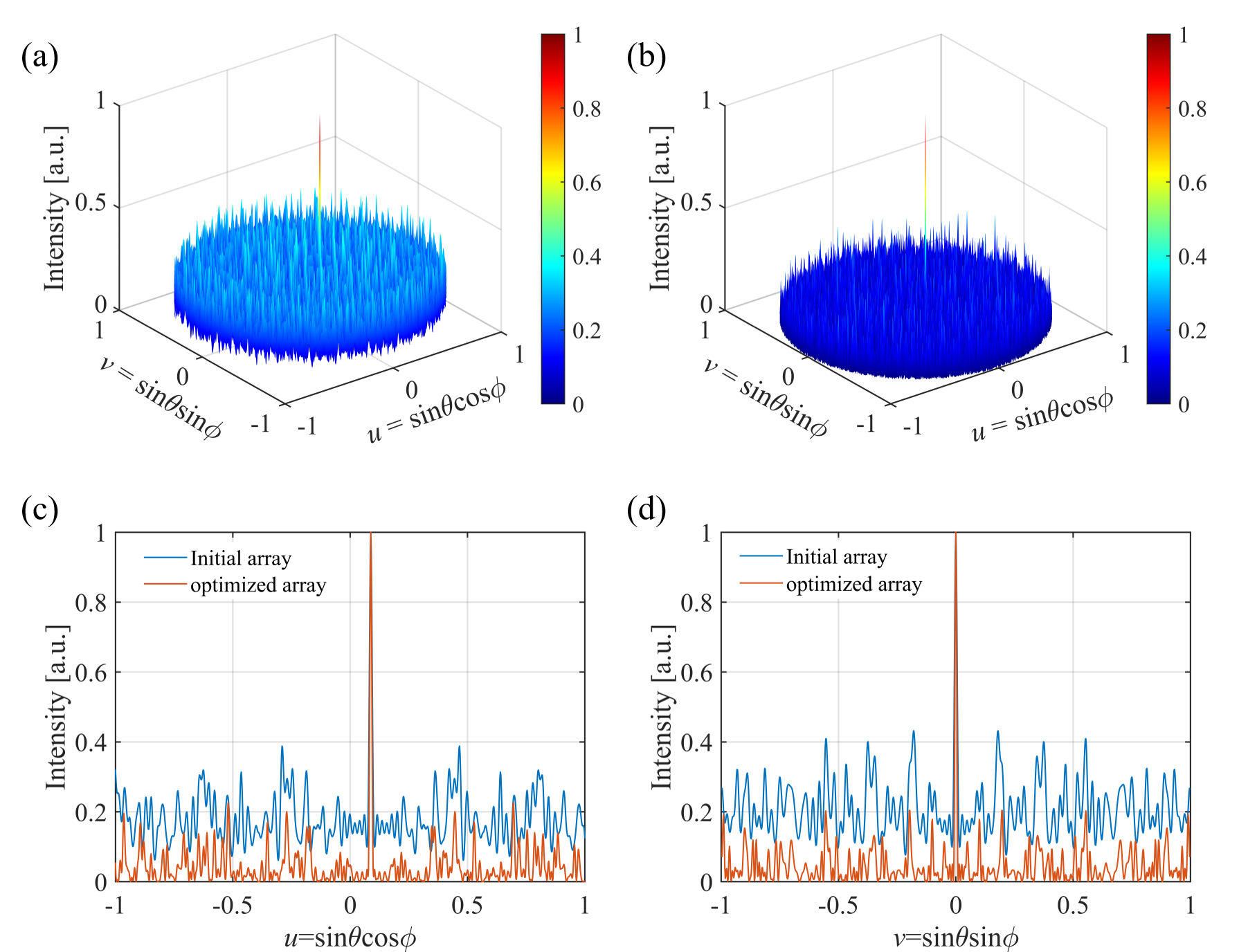


**Figure S5:** Simulation results for the 32-element sparse circular array. (a) 2-D far-filed pattern for the initial array. (b) 2-D far-filed pattern for the optimized array. 1-D cross-sections of the intensity pattern along (c) *u* and (d) *v* directions with initial array and optimized array.

The ratio of the power of the 3-dB main lobe and the total power in the full FOV for the array before and after optimization are also simulated and compared. Here, we propose to apply the Monte Carlo method to estimate this value. For the total energy, first generate *N* random particles inside a cylinder with the base radius of 1 and the height of 1. Record the number of random particles *N’* that fall within the 2-D far-field intensity surface, then the total energy is calculated by *N’*/ *N*·*V*_cylinder_ = *N’*/ *N*·π_._ Similarly, the calculation of the 3-dB main lobe power is also finding the corresponding cylindrical bottom of the main lobe, generating a certain number of random particles in a cylinder with the height of 1, and calculating the 3-dB main lobe power based on the number of particles falling within the 3-dB surface. The simulation in Figure S6(a)-S6(d) show the proportion of the main lobe energy to the total energy for the initial distribution and the optimized distribution is 2.07×10^-4^ and 5.59×10^-4^, respectively.

**
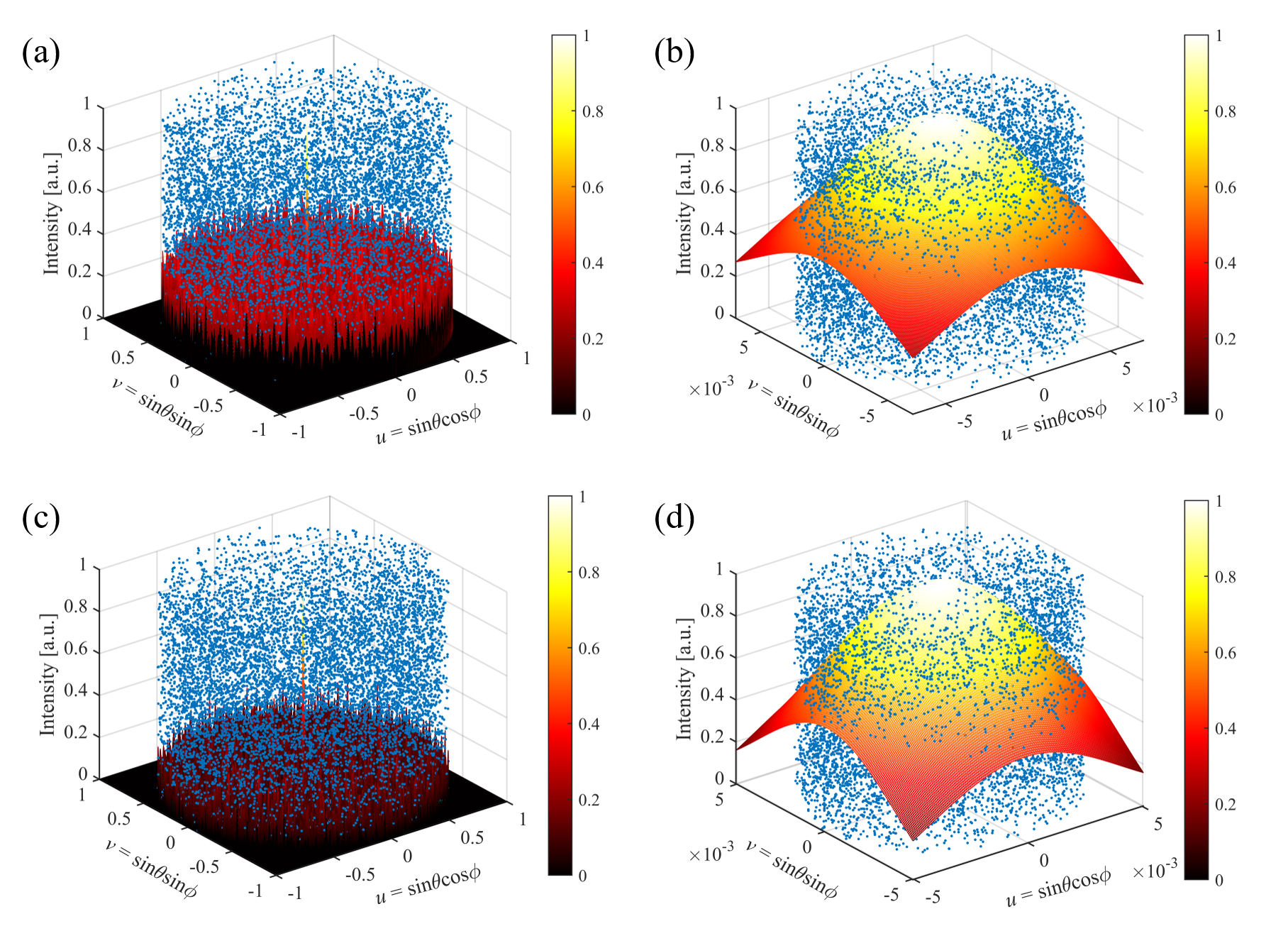
**

**Figure S6:** Simulation results for the proportion of the main lobe energy to the total energy. For the initial array, calculation of (a) the total power in full FOV and (b) the 3-dB main lobe power. For the optimized array, calculation of (c) the total power in full FOV and (d) the 3-dB main lobe power.

**3. Principle of golden section search method**

The modified rotating electric field vector method (mREV) algorithm has a good performance in achieving better algorithm convergence. However, it is necessary to traverse the voltage values of the 0 to 2π modulation phase on each channel with a fixed step size, the process can be very time-consuming. Fortunately, as shown in Figure 3(a), The *E* field of each element is regarded as a vector, the phase of one element is varied from 0 to 2π at a time and simultaneously detect the light intensity at the target direction, the rotation stops when the intensity reaches the largest since there is only one maximum intensity at this direction in the far-field during the 0 to 2π phase change of OPA single channel output light. This type of problem is suitable for solving with the golden section search method.

The principle of the gold section search method is shown in Figure S7. For unimodal functions with interval [*a*, *b*], first we take the two middle trial points c and d of the interval to satisfy the golden ratio, which is defined as: *c* = *b*–0.618(*b*–*a*), *d* = *a*+0.618(*b*–*a*). Then we compare the output value *f*(*c*) and *f*(*d*): if *f*(*c*) > *f*(*d*), the interval end point *b* of the next iteration is assigned to *d*, and at this time the trial point *d* satisfies that *d* equals to *c*. It is only necessary to recalculate the coordinates of point *c* and compare *f*(*c*) in the current round with *f*(*c*) in the previous round (that is, *f*(*d*) in the current round). The process is similar to the above when *f*(*c*) ≤ *f*(*d*). The interval length *b*–*a* is reduced by 0.618 times after each iteration, and when interval length is less than the convergence accuracy *ξ*, the algorithm stops.


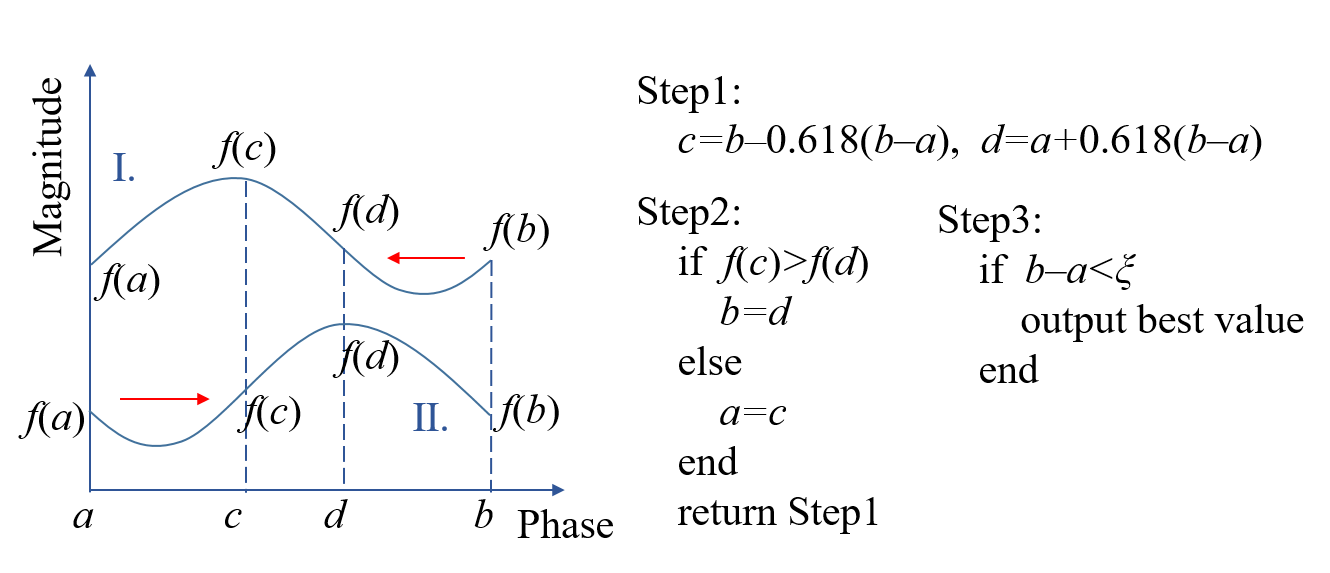


**Figure S7:** Schematic diagram of gold section search method.

**4. Characterization of the phase modulator**

We have fabricated and tested the Mach-Zehnder interferometer (MZI) structure to characterize the performance of the phase modulator of our OPA. As shown in Figure S8(a), the power efficiency of the phase modulator is about 22.3 mW/π. Figure S8(b) shows the measured voltage and current value of the phase modulator and the linear fitting curve. The resistance value fluctuates to a certain extent during the change of voltage from 1 V to 6 V and the fitting resistance is approximately 407.9 Ω.


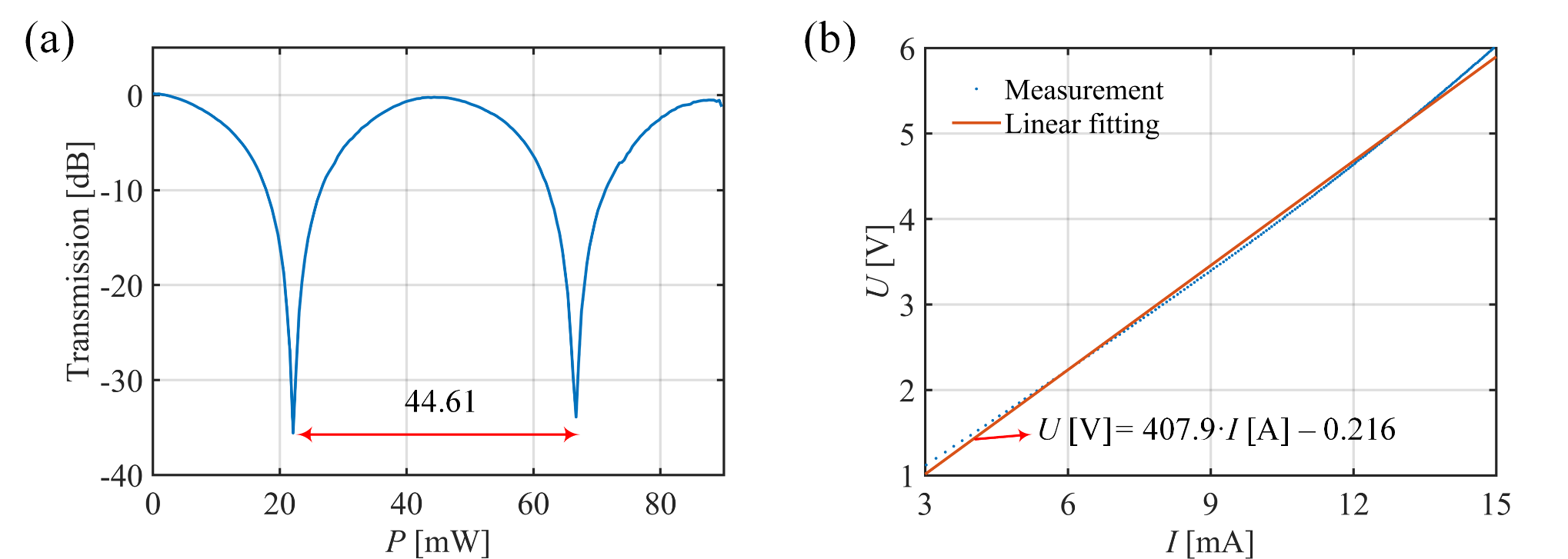


**Figure S8:** Measurement results for the phase modulator. (a) Optical transmission various with the applied power of the MZI. (b) Voltage various with current curve of MZI.

**5. Calculation of the calibrated voltage for the particular steering angle**

In this case of sparse distribution of antenna, the phase factor would be no longer linear like uniform linear array or uniform rectangular array. While, if we acquire calibrated voltage for an arbitrary steering angle, the original phase distribution without calibration can be solved out approximately. Then the calibrated voltage of each element for the particular steering angle can be calculated with the power corresponding to 2π phase shift and the resistance of phase shifters.

Firstly, we assume that all antennas have unequal lengths and 32 initial phases are generated randomly. The GSS-REV algorithm is applied to calibrate the steering angle of (0, 0), with the calibrated voltage the required phase of the *i*-th element for the particular steering angle (*u_x_*, *u_y_*) is expressed as:

where $r_{i}$ is the radius of the ring where the *i*-th element is located, $\theta_{i}$ is the angular position of the *i*-th element, $V_{i}$ is the calibrated voltage of the *i*-th element for the steering angle of (0, 0), $R$ is the average resistance of the phase shifters and $P_{2\pi}$ is the required power for the phase shift of 2π. Then the calibrated voltage of each element for the (*u_x_*, *u_y_*) angle is calculated by:

The simulation is conducted as follows. A Gaussian distribution error within the range of 0.1 % peak main lobe intensity is introduced in far-field optical power measurement data to simulate the effect of power measurement inaccuracy, considering the non-uniformity of the manufacturing and the issue of thermal crosstalk, the resistance and the power corresponding to 2π phase shift for 32 elements are set as 32 random numbers, respectively, which is (1±2.5%)×407.9 Ω and (1±2.5%)×44.6 mW. Figure S9(a) shows the 32 calibrated voltages for the angle of (0, 0) and the calculated voltages for the particular angle of (0,1, 0.1) and Figure S9(b) is the far-filed pattern of this steering angle. As shown in Figure S9(c) and S9(d), the cross-sections of the intensity pattern along *u_x_* and *u_y_* directions with error free, calculation and calibration are presented, among which the side lobes have a similar level, verifying the good phase accuracy through calculation.


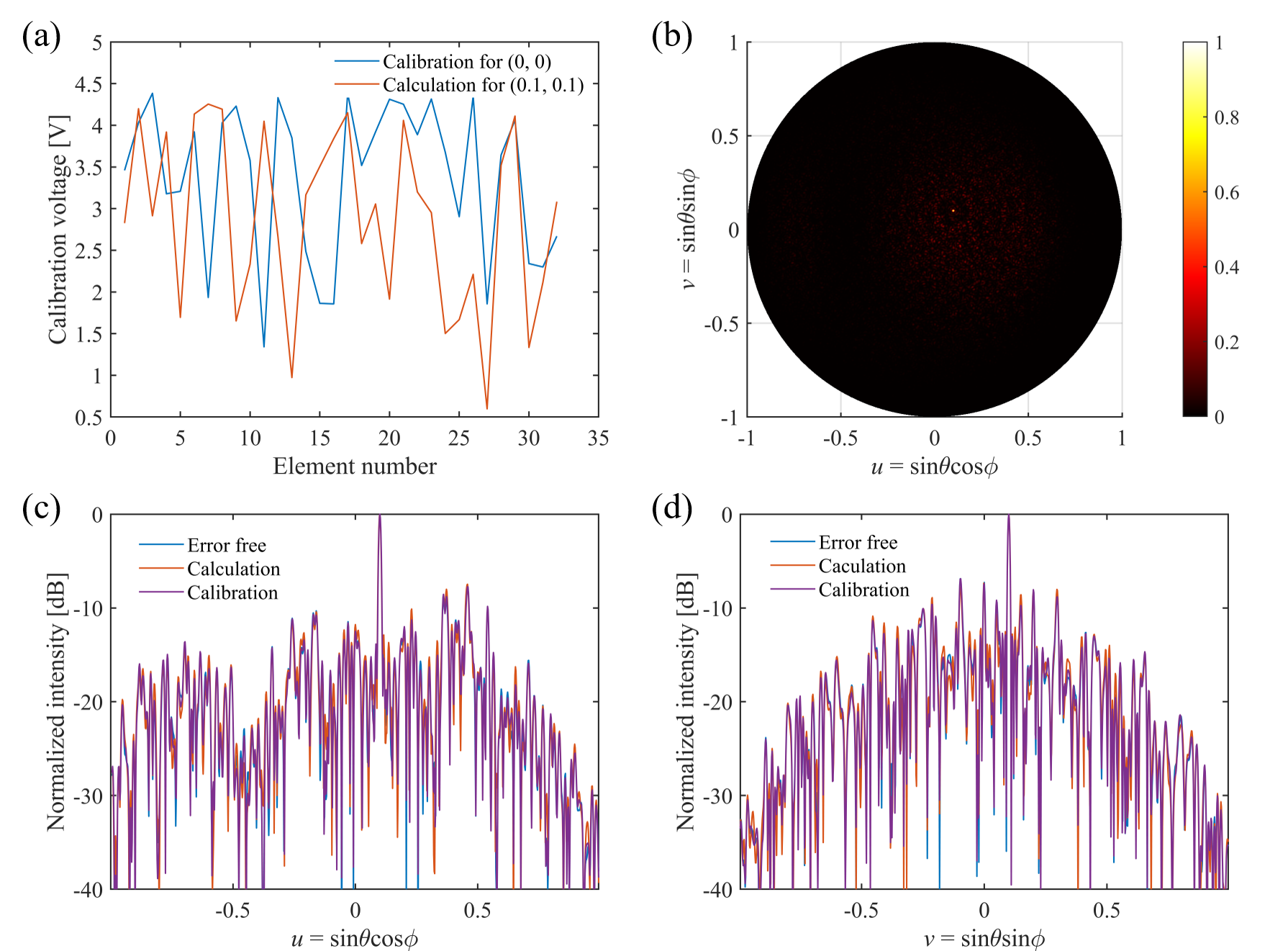


**Figure S9:** (a) Calibrated voltages for (0, 0) and Calculated voltages for (0.1, 0.1). (b) Far-field intensity pattern on the u-v plane with the main lobe of (0.1, 0.1). (c) Far-field intensity pattern on the u-v plane with the main lobe of (0.1, 0.1). 1-D cross-sections of the intensity pattern along (c) *u_x_* and (d) *u_y_* directions with error free, calculation and calibration.

**6. Range measurement based on the FMCW system**

Figure S10 shows the transmitted (blue waveform) and received (green waveform) optical frequency chirped signal in the FMCW system with the time-delayed for a moving target, the frequency modulation period and the extent of the transmitter optical frequency modulation respectively defined as Δ*t*, *T*, *B*.


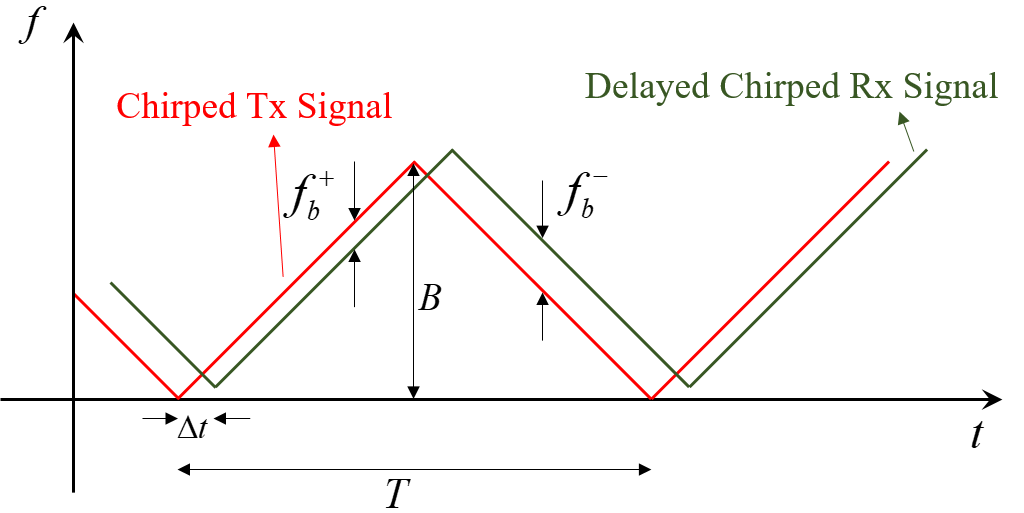


**Figure S10:** Schematic of a typical FMCW LiDAR laser modulation ramp (red) and time-delayed received signal (green) waveforms, assuming the target is moving towards the system.

Considering the target is stationary relative to the transmitter in our measurement system, the theoretical relationship between range and beat frequency for FMCW LiDAR is written as: $R/f_{b}=c\cdot T/\left(4B\right)$ [S1], here *R* is the distance between target and transmitter, *f_b_* is the frequency of beat frequency signal, *c* is the speed of light. In our demonstration, the simulation slope of range vs. FMCW signal beat frequency is calculated to be 1.116 m/MHz and the fitting slope is 1.140 m/MHz. The difference between simulation and fitting slope value is approximately 2.15%.

**References:**

1. K. Sayyah, R. Sarkissian, P. Patterson, et al., "Fully Integrated FMCW LiDAR Optical Engine on a Single Silicon Chip," *J*. *Lightwave* *Technol*., vol. 40, pp. 2763-2772, 2022.
